# Syncope as the Sole Manifestation of a Saddle Pulmonary Embolism in a Hemodynamically Stable Patient

**DOI:** 10.7759/cureus.102660

**Published:** 2026-01-30

**Authors:** Joshua Dodge, Eugene Kang

**Affiliations:** 1 Emergency Medicine, University of Nevada Las Vegas School of Medicine, Las Vegas, USA; 2 Emergency Medicine, University Medical Center of Southern Nevada, Las Vegas, USA

**Keywords:** emergency medicine imaging, pulmonary embolism rule-out criteria (perc), pulmonary embolism with syncope, pulmonary thrombectomy, saddle pulmonary embolism, syncope, wells pe score

## Abstract

Syncope is a common emergency department presentation with many potential causes. Pulmonary embolism (PE) typically presents with chest pain, dyspnea, and abnormal vital signs, but has been reported without typical signs or symptoms. A 77-year-old man experienced sudden syncope while carrying water cartons. He denied prodromal symptoms or cardiopulmonary complaints. His history included hypertension, chronic kidney disease, glaucoma, and hyperlipidemia. However, he denied major risk factors for PE. On arrival, he was hemodynamically stable, with only a forehead abrasion on exam. Labs revealed thrombocytopenia (89 × 10³/μL) (reference range: <150 x 10³/μL), elevated troponin (120 ng/L) (reference range: <40 ng/L), and B-type natriuretic peptide (BNP) (374 pg/mL) (reference range: <125 pg/mL). Electrocardiogram revealed new right heart strain, while the chest radiograph was unremarkable. CT pulmonary angiography confirmed acute saddle PE with a large clot burden. He was anticoagulated and admitted, but developed hemodynamic instability within 24 hours, requiring catheter-directed thrombectomy. He stabilized and was discharged on hospital day 6, on oral anticoagulation. This case underscores that PE can present as isolated syncope, highlighting the importance of considering subtle clinical and laboratory clues.

## Introduction

Syncope accounts for approximately 0.3% of emergency department (ED) visits and arises from a wide variety of etiologies, including cardiogenic, orthostatic, vasovagal, neurogenic, and pulmonary causes [1,2]. Pulmonary embolism (PE) is a particularly challenging consideration, given its potentially fatal consequences if missed and the risks associated with overtesting. D-dimer assays, while highly sensitive, are non-specific and frequently elevated in patients without PE, limiting their diagnostic utility [3]. Computed tomography pulmonary angiography (CTPA) remains the gold standard for diagnosis but carries risks of radiation exposure and contrast-related complications, and treatment of clinically insignificant emboli with anticoagulation may expose patients to unnecessary bleeding risk [4].

Clinical decision-making tools such as the Wells score and the Pulmonary Embolism Rule-Out Criteria (PERC) have been validated to guide the evaluation of suspected PE [5]. However, given that PE is a rare cause of syncope and clinical gestalt plays a major role in evaluation of PE, many clinicians may opt to not to perform laboratory or imaging evaluation of PE in patients without other evidence of PE, such as chest pain, dyspnea, vital sign abnormalities, or other major risk factors for PE. In part, this is because overtesting for PE can lead to harm. Recent studies have investigated how to risk-stratify patients for PE in the context of syncope. They suggest that patients with normal vital signs and without chest pain, dyspnea, or major risk factors may not require additional testing [6]. Here, we present a case of PE manifesting as isolated syncope in a patient with no major risk factors, normal vital signs, and no cardiopulmonary complaints.

## Case presentation

A 77-year-old man presented to the ED after a syncopal event. He reported carrying cartons of water into his garage when he abruptly lost consciousness, awakening moments later to bystanders calling paramedics. His estimated downtime was less than a few minutes. The patient noted urinary incontinence, but neighbors denied witnessing seizure-like activity. The patient had no prior seizure history. He denied prodromal symptoms, chest pain, palpitations, shortness of breath, dizziness, headache, visual or auditory changes, focal weakness, or sensory deficits.

Past medical history included hypertension, stage 3 chronic kidney disease (not on dialysis), glaucoma, and hyperlipidemia. He denied prior venous thromboembolism, malignancy, recent hospitalization or surgery, immobility, hormone therapy, or hemoptysis. He had no history of recent travel.

On arrival, vital signs were unremarkable: heart rate 77 bpm, blood pressure 141/87 mmHg, respiratory rate 14 breaths/min, oxygen saturation 99% on room air, and temperature 37°C. The patient appeared generally well, conversational, and in no acute distress. Examination was significant for a left forehead abrasion and hematoma. The patient had a normal cardiopulmonary exam and neurovascular exam with no stigmata of deep vein thrombosis (DVT).

**Figure 1:**
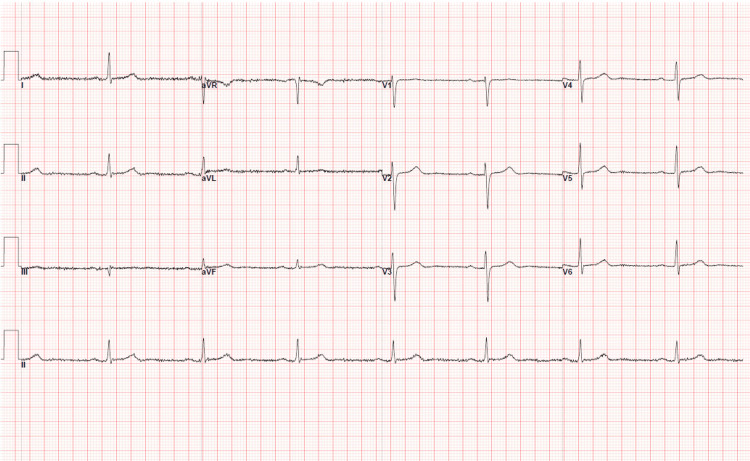
Patient’s baseline EKG prior to presentation EKG, electrocardiogram.

**Figure 2:**
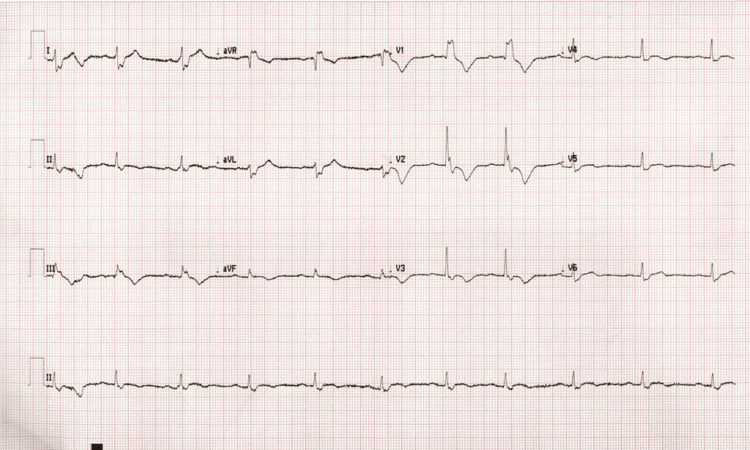
Patient’s EKG in the emergency department at presentation EKG, electrocardiogram.

Laboratory testing showed normal complete blood count and comprehensive metabolic panel, aside from chronic renal insufficiency and a newly developed thrombocytopenia (platelet count 89 × 10³/μL) (reference range: <150 x 10³/μL) down from 154 × 10³/μL four months ago. Troponin was elevated at 120 ng/L (reference range: <40 ng/L) and B-type natriuretic peptide (BNP) was elevated at 374 pg/mL (reference range: <125 pg/mL), both without previous comparisons. Creatine kinase was normal. The chest radiograph was unremarkable. Electrocardiogram demonstrated new right heart strain compared with a prior study (Figures 1, 2).

**Figure 3:**
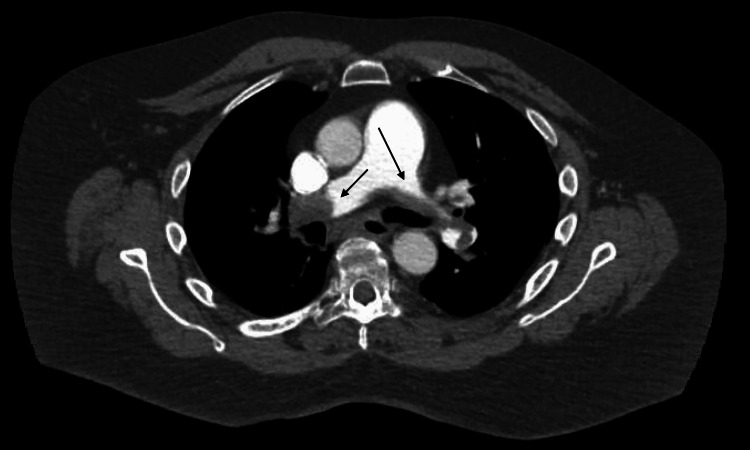
CTA axial view of chest demonstrating saddle pulmonary embolism with a large clot burden Black arrows point to the thromboembolisms in the pulmonary artery. CTA, computed tomography angiography.

**Figure 4:**
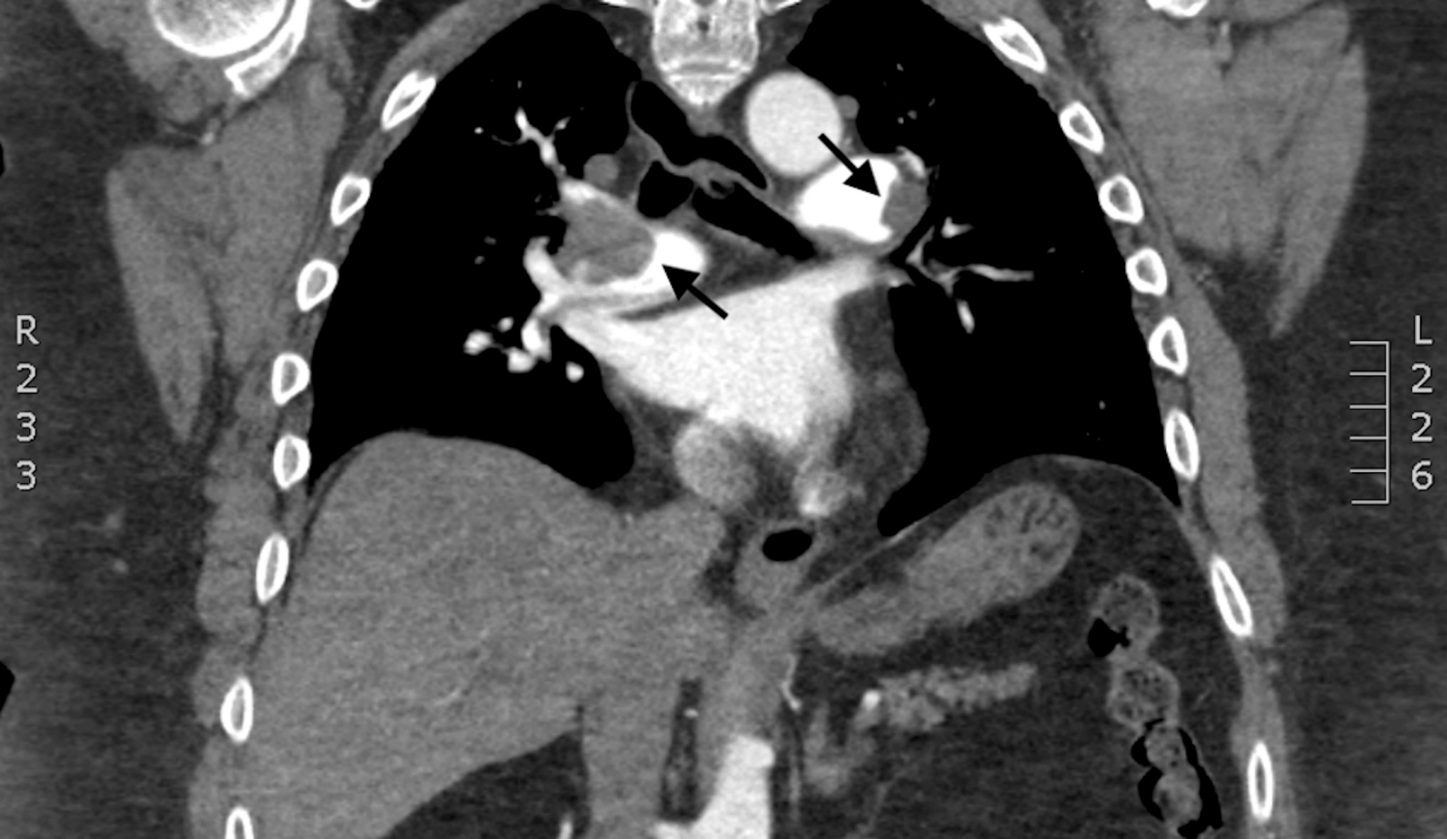
CTA coronal view of chest demonstrating saddle pulmonary embolisms with a large clot burden Black arrows point to the thromboembolisms in the pulmonary artery. CTA, computed tomography angiography.

Given the presence of right heart strain and elevated cardiac biomarkers despite clinical stability, PE was suspected. CT of the pulmonary arteries confirmed the diagnosis of bilateral PEs with right heart strain (Figures 3, 4).

Interventional radiology was consulted, and anticoagulation with intravenous heparin was initiated. The patient was admitted to the intensive care unit for monitoring. Throughout the entirety of the ED course, the patient had normal vital signs. Within 24 hours, he developed hemodynamic collapse, requiring emergent catheter-directed thrombectomy, which was successful. He was neurologically intact and was discharged on oral anticoagulation in stable condition.

## Discussion

This case illustrates the diagnostic challenge of PE presenting as syncope in a patient without classic symptoms, major risk factors, or abnormal vital signs. The PESIT trial demonstrated that as many as 17.3% of patients who presented to the ED with syncope had a diagnosis of PE [7]. Of these patients, 24.7% of them had no overt clinical signs or symptoms of PE. On further subgroup analysis, it appears that roughly two-thirds of these patients had segmental or subsegmental emboli and approximately one-third had a lobar or more proximal clot. None had right ventricular strain when an echo was performed, suggesting that these may have been “silent” PEs that were found incidentally and without clinical relevance. Follow-up studies also suggest that the rates of syncope-related PE as described in the PESIT trial may be grossly overestimated, with rates closer to 0.5-1.3% [8,9]. In this smaller cohort of patients, most presented to the ED with symptoms of PE or abnormal vital signs. This makes our case of syncope as a sole manifestation of PE in an otherwise hemodynamically stable patient so unique. 

This case underscores the need to consider PE in syncopal patients without other typical signs or symptoms of PE. In this instance, the new finding of right heart strain on electrocardiogram (EKG), combined with elevated cardiac biomarkers, provided the crucial diagnostic clue that prompted further imaging.

Additionally, the progression of this patient’s illness highlights the dynamic nature of PE. Although initially stable, he developed hemodynamic instability, requiring intervention within 24 hours. This clinical trajectory reinforces the importance of close monitoring, even in patients who appear stable at presentation. Early recognition and anticoagulation likely contributed to the favorable outcome in this case, and escalation to thrombectomy was appropriately pursued when the patient deteriorated. Finally, this case emphasizes the importance of maintaining a broad differential diagnosis in elderly patients presenting with syncope. While cardiac arrhythmias, neurogenic causes, or orthostatic hypotension may be more common explanations, PE should remain in the differential, particularly when subtle signs of right heart strain or biomarker elevation are present.

## Conclusions

PE can present solely as syncope without chest pain, dyspnea, or vital sign abnormalities. This case demonstrates the limitations of relying exclusively on risk stratification tools and highlights the need for clinical vigilance when atypical but potentially life-threatening conditions are possible. In elderly patients presenting with unexplained syncope, subtle findings such as new right heart strain on EKG or elevated cardiac biomarkers should prompt consideration of PE, even in the absence of traditional risk factors. Early recognition, close monitoring, and timely intervention are critical to improving outcomes in such presentations.
